# The role of respiratory co-infection with influenza or respiratory syncytial virus in the clinical severity of COVID-19 patients: A systematic review and meta-analysis

**DOI:** 10.7189/jogh.12.05040

**Published:** 2022-09-17

**Authors:** Bingbing Cong, Shuyu Deng, Xin Wang, You Li

**Affiliations:** 1 Department of Epidemiology, School of Public Health, Nanjing Medical University, Nanjing, 211166, China; 2 Department of Biostatistics, School of Public Health, Nanjing Medical University, Nanjing, China

## Abstract

**Background:**

With the easing of COVID-19 non-pharmaceutical interventions, the resurgence of both influenza and respiratory syncytial virus (RSV) was observed in several countries globally after remaining low in activity for over a year. However, whether co-infection with influenza or RSV influences disease severity in COVID-19 patients has not yet been determined clearly. We aimed to understand the impact of influenza/RSV co-infection on clinical disease severity among COVID-19 patients.

**Methods:**

We conducted a systematic literature review of publications comparing the clinical severity between the co-infection group (ie, influenza/RSV with SARS-CoV-2) and mono-infection group (ie, SARS-CoV-2), using the following four outcomes: need or use of supplemental oxygen, intensive care unit (ICU) admission, mechanical ventilation, and deaths. We summarized the results by clinical outcome and conducted random-effect meta-analyses where applicable.

**Results:**

Twelve studies reporting a total of 7862 COVID-19 patients were included in the review. Influenza and SARS-CoV-2 co-infection were found to be associated with a higher risk of ICU admission (five studies, odds ratio (OR) = 2.09, 95% confidence interval (CI) = 1.64-2.68) and mechanical ventilation (five studies, OR = 2.31, 95% CI = 1.10-4.85). No significant association was found between influenza co-infection and need/use of supplemental oxygen or deaths among COVID-19 patients (four studies, OR = 1.04, 95% CI = 0.37-2.95; 11 studies, OR = 1.41, 95% CI = 0.65-3.08, respectively). For RSV co-infection, data were only sufficient to allow for analyses for the outcome of deaths, and no significant association was found between RSV co-infection and deaths among COVID-19 patients (three studies, OR = 5.27, 95% CI = 0.58-47.87).

**Conclusions:**

Existing evidence suggests that co-infection with influenza might be associated with a 2-fold increase in the risk for ICU admission and for mechanical ventilation among COVID-19 patients whereas evidence is limited on the role of RSV co-infection. Co-infection with influenza does not increase the risk of death in COVID-19 patients.

**Registration:**

PROSEPRO CRD42021283045.

Coronavirus disease 2019 (COVID-19), caused by the severe acute respiratory syndrome coronavirus 2 (SARS-CoV-2), remains a global pandemic and public health concern [1]. Despite the rollout of vaccination programs and implementation of non-pharmaceutical interventions (NPIs), the number of infected cases kept rising rapidly, particularly after the emergence of the delta and (more recently) the omicron variant. As of March 17, 2022, there have been over 460 million COVID-19 cases and over 6 million deaths globally [2].

Influenza and respiratory syncytial virus (RSV) are the two most common respiratory viruses, affecting mainly young children and older adults, especially in low- and middle-income countries (LMICs) [3-7]. Globally, it is estimated that in 2016, influenza and RSV were associated with 39 and 25 million acute lower respiratory infection episodes, and 58 000 and 77 000 deaths, respectively [8]. In most temperate regions, influenza and RSV normally circulate in autumn and winter months [9]. During the COVID-19 pandemic, the activity of both viruses was low early on due to the large-scale implementation of NPIs, but the resurgence of RSV epidemics was observed in several countries since late 2020 [10], followed by the resurgence of influenza epidemic in the winter of 2021 [11]. While the re-emergence of influenza and RSV will surely put pressure on health care providers already over-stretched in response to the COVID-19 pandemic, the effect of a co-infection with influenza or RSV on the clinical severity of individual COVID-19 patients is yet unknown.

In this study, we aimed to systematically and critically review the existing evidence on the impact of co-infection with influenza or RSV on disease severity in COVID-19 patients.

## METHODS

### Search strategy and selection criteria

This systematic review was reported according to the Preferred Reporting Items for Systematic Reviews and Meta-Analyses (PRISMA; Text S1 in the **Online Supplementary Document**) and Synthesis without meta-analysis in systematic reviews (SWiM; Text S2 in the **Online Supplementary Document**) reporting guidelines. The protocol of this review was registered in International Prospective Register of Systematic Review (PROSEPRO) with registration number CRD42021283045. We searched the following electronic databases:MEDLINE, EMBASE, Web of Science, the WHO COVID-19 Global literature on coronavirus disease database, China National Knowledge Infrastructure (CNKI), WanFang, CqVip, and Sinomond for relevant publications from January 1, 2020 to December 31, 2021 using a tailored search strategy (Text S3 in the **Online Supplementary Document**). No language restrictions were applied. The reference lists of eligible studies were also examined.

#### Inclusion criteria

Population-based studies reporting any laboratory-confirmed co-infections with influenza or RSV in COVID-19 patients were included, providing that at least one of the following outcomes was reported separately in co-infection group (ie, SARS-CoV-2 and influenza/RSV) and mono-infection group (ie, SARS-CoV-2 mono-infection group should test negative for influenza and RSV): need or use of supplemental oxygen, ICU admission, mechanical ventilation (including invasive and non-invasive ventilation) and deaths.

#### Exclusion criteria

The following studies were excluded: 1) studies that focused on reporting nosocomial infections, or 2) studies that only included patients with comorbidities (eg, patients with chronic obstructive pulmonary disease, or patients infected with human immunodeficiency virus), or 3) reviews or studies reporting data that were previously reported by another study (in which case only the primary studies were considered for inclusion).

### Systematic literature review

Two reviewers (BC and SD) independently screened titles, abstracts and full texts of retrieved records and extracted data using a tailored data extraction template comprising two parts: the first part collected study-level characteristics such as the study location, period, number of subjects, age of subjects, statistical method, disease severity outcomes reported, clinical specimens, viral diagnostic techniques and so on; the second part collected data on the clinical outcomes by mono- infection group and co-infection group. Any discrepancies during data screening and extraction were resolved among YL, BC, and SD

### Quality assessment

Two reviewers (BC and SD) independently assessed all included studies for quality. A modified Critical Appraisal Skills Programme (CASP) checklist for cohort studies was used for the quality assessment [12]. The modified questionnaire contained the following seven questions: 1) Did the study address a clearly focused issue? 2) Were the subjects recruited in an acceptable way? 3) Was the exposure accurately measured to minimize bias? 4) Was the outcome accurately measured to minimize bias? 5) Have the authors used multivariable analysis method to adjust for confounders? 6) Can the results be applied to the local population? 7) Do the results of this study fit with other available evidence? The questionnaire contained seven questions and answer to each of the questions could be “Yes”, “No”, or “Can’t tell”, corresponding to 1, 0, and 0 points, respectively. We calculated the overall score for each study after assessing each criterion as listed above. Studies were defined as “high quality” (7 points), “moderate quality” (6-5 points), and “low quality” (≤4 points). Any discrepancies during quality assessment were resolved among YL, BC, and SD

### Data analysis

A narrative synthesis was conducted for all outcomes of interest. The outcomes were compared between the mono-infection group and the co-infection group. A random-effect meta-analysis of the corresponding odds ratios was conducted if three or more studies were available per comparison (ie, influenza and SARS-CoV-2 co-infection vs SARS-CoV-2 mono-infection, and RSV and SARS-CoV-2 co-infection vs SARS-CoV-2 mono-infection). The choice of conducting a random-effect meta-analysis (rather than fixed-effect meta-analysis) was based on the anticipation that populations included in the studies differed by region, age, study period (in relation to the COVID-19 pandemic), clinical specimens, and diagnostic methods. We applied a continuity correction of 0.5 if no one had severity outcomes in any group [13]. This allowed odds ratios (ORs) to be calculated and enabled inclusion within subsequent meta-analyses. When ORs could be obtained both from univariate analysis and multivariate analysis in a report, the latter was included in the meta-analysis. For influenza and SARS-CoV-2 co-infection, the subgroup analysis was conducted by influenza type (ie, influenza A and influenza B) if data allowed. Sensitivity analyses excluding studies with small sample sizes (defined as ≤5 subjects in any of the mono-infection and co-infection groups) and excluding those low-quality studies were performed. Symmetry of funnel plot and Egger’s regression method were used to evaluate the presence of small study effects [14]. Heterogeneity was evaluated by *I^2^* values; *I^2^* values of >50% and >75% suggested moderate and high heterogeneity, respectively [15]. All statistical analyses and data visualizations were performed with R (version 4.1.0).

## RESULTS

### Review process

**Figure 1:**
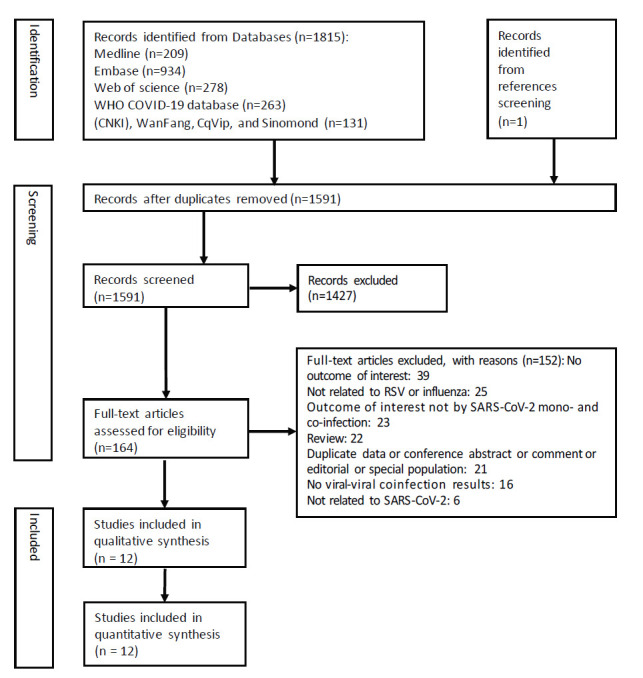
PRISMA diagram for selection of studies.

After elimination of duplicates, 1591 records were assessed by title and abstract; 164 records were further assessed by full text. A total of 12 studies were included (**Figure 1**).

### Characteristics of included studies

Of the 12 studies included, 11 [16-26] investigated the role of influenza co-infection with SARS-CoV-2 in the outcomes of interest, and three [22,23,27] investigated the role of RSV co-infection with SARS-CoV-2. The included studies represented 955 laboratory-confirmed COVID-19 patients co-infected with influenza or RSV and 6907 patients infected with SARS-CoV-2 mono-infection. All studies reported deaths as one of the outcomes of interest. Four studies reported the outcome of need or use of supplemental oxygen; six reported on mechanical ventilation and six reported on ICU admission.

All studies except one [19] (mixed settings, ie, inpatients, emergence department and outpatients) were conducted in inpatient settings. Subject age varied greatly across the included studies and was reported in various statistical forms (eg, frequency by age group, mean and standard deviation, median, etc.). The total number of SARS-CoV-2 patients co-infected with influenza/RSV and mono-infected patients per study ranged from 22 to 4501. Nine studies were mainly conducted in Asia, six of which were from China [16,17,21-23,25]. For viral detection method, SARS-CoV-2 was detected using PCR for all studies whereas influenza and / or RSV co-infection was confirmed by PCR, serological testing, or antigen assays. The quality assessment of included studies is provided in Table S1 in the **Online Supplementary Document**. Four studies [16,17,19,25] were assessed as high-quality and they used multivariate statistical methods to account for common confounders such as age, sex, and comorbidities. The basic characteristics of the included studies are available in **Table 1**.

**Table 1:** Characteristics of included studies

Study	Study period	Country	Setting	Age of subjects	Number of subjects	Specimen (s)	Diagnostic method (s)	O2	MV	ICU	Death	Score, quality
Wu, 2020 [16]	January 2020 – April 2020	China	IP	Mean (SD), majority (88.8%) = 57.1 (15.7) y	1386	NPS/BLF/sputum/BAF/blood	RT-PCR/IFA	+	−	−	+	7, high
Tong, 2020 [17]	February 2020 – March 2020	China	IP	Median (IQR) = 65.0 (48.5, 70.0) y	140	Throat swab/blood	RT-PCR/IFA	−	+	−	+	7, high
Alosaimi, 2021 [18]	2020	Saudi Arabia	IP + ICU	All ages	31	NPS	RT-PCR/quantitative PCR	−	−	+	+	4, low
Stowe, 2021 [19]	January 2020 – April 2020	UK	IP + ER + OP	All ages	4501	NPA/throat swab/tracheal secretion/nasal swab	RT-PCR/quantitative PCR	−	+	+	+	7, high
Takahashi, 2021 [20]	March 2020 – May 2020	America	IP	Adults (>18y)	920	NA	RT-PCR	−	−	−	+	5, moderate
Zhu, 2020 [21]	January 2020 – February 2020	China	IP	All ages	22	Throat swab	RT-PCR	−	−	−	+	5, moderate
Zhang, 2020 [22]	January 2020 – February 2020	China	IP	Median (IQR) = 33.00 (10.00-94.25) m	29	NPS / throat swab	RT-PCR	+	+	+	+	4, low
Li, 2021 [23]	January 2020 – February 2020	China	IP	Mean (SD), majority (66.7%) = 59.88 (7.63) y	58	Sputum/throat swab	RT-PCR/culture/DFA	−	−	−	+	5, moderate
Agarwal, 2021 [24]	August 2020 – December 2020	India	IP	Median (IQR) = 58 (48-65) y	101	Upper respiratory tract samples	RT-PCR	−	+	+	+	6, moderate
Zheng, 2021 [25]	January 2020 – April 2020	China	IP	Adults (>18y)	285	NPS/sputum/BLF	RT-PCR	+	+	+	+	7, high
Akhtar, 2021 [26]	March 2020 – December 2020	Bangladesh	IP	Median (IQR), 28 (1.2–53) y	285	NPS/OPS	RT-PCR	+	−	−	+	6, moderate
Alvares, 2021 [27]	March 2020 – September 2020	Brazil	IP	Range, <2 y	32	NPS	RT-PCR/CL	−	+	+	+	6, moderate

ER – emergency department, IP – inpatient, OP – outpatient, NPS – nasopharyngeal swab, OPS – oropharyngeal swab, BLF – bronchoalveolar lavage fluid, BAF – bronchial aspiration fluid, NPS – nasopharyngeal aspirate, NA – not available, RT-PCR – reverse transcription polymerase chain reaction, DFA – direct immunofluorescence assay, IFA – indirect immunofluorescence assay, CL – chemiluminescence, CIA – chromatographic immunoassay, number of subjects – the total number of SARS-CoV-2 coinfected with influenza/RSV and mono-infected patients, O2 – oxygen, MV – mechanical ventilation, ICU – intensive care unit, IQR – interquartile range, SD – standard deviation, y – years, m – month

### Co-infection and risk of need or use of supplemental oxygen

Four studies [16,22,25,26] reported the need or use of supplemental oxygen as an outcome (four on influenza and one [22] on RSV co-infection).

**Figure 2:**
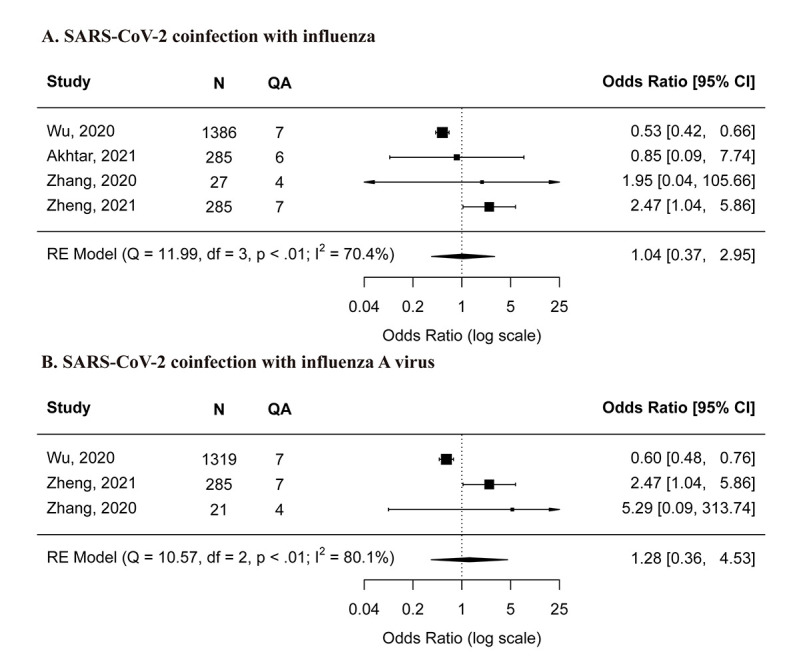
Comparison of risk for need or use of supplemental oxygen between SARS-CoV-2 mono-infection and A) SARS-CoV-2 co-infection with influenza, B) SARS-CoV-2 co-infection with influenza A virus. **Panel A** – SARS-CoV-2 co-infection with influenza. **Panel B** – SARS-CoV-2 co-infection with influenza A virus. N – the total number of SARS-CoV-2 coinfected and mono-infected patients, QA – the score of quality assessment.

Our meta-analysis showed that SARS-CoV-2 co-infection with influenza was not associated with an increased need or use of supplemental oxygen compared with SARS-CoV-2 mono-infections (OR = 1.04, 95% confidence interval (CI) = 0.37-2.95) (**Figure 2**, panel A). When excluding studies with small sample size or low-quality studies, the meta-estimates did not differ substantially from the main analyses (Figure S1, panel A and Figure S2, panel A in the **Online Supplementary Document**). SARS-CoV-2 co-infection with influenza A virus was also not observed to be associated with increased need or use of supplemental oxygen (OR = 1.28, 95% CI = 0.36-4.53) (**Figure 2**, panel B). Two high-quality studies showed contrasting findings: one study [16] showed SARS-CoV-2 co-infection with influenza A virus was associated with a decreased need or use of supplemental oxygen (OR = 0.61, 95% CI = 0.48-0.76) whereas no such difference was observed on SARS-CoV-2 co-infection with influenza B virus (OR = 0.97, 95% CI = 0.56-1.67); the other study [25] showed that SARS-CoV-2 co-infection with influenza was associated with increased need or use of supplemental oxygen (OR = 2.47, 95% CI = 1.04-5.86).

Only one study [22] provided data on SARS-CoV-2 co-infection with RSV; however, none of the included patients in that study required supplemental oxygen.

### Co-infection and risk of mechanical ventilation

Six studies [17,19,22,24,25,27] reported mechanical ventilation as an outcome (all but one [27] on influenza and two studies [22,27] on RSV co-infection).

**Figure 3:**
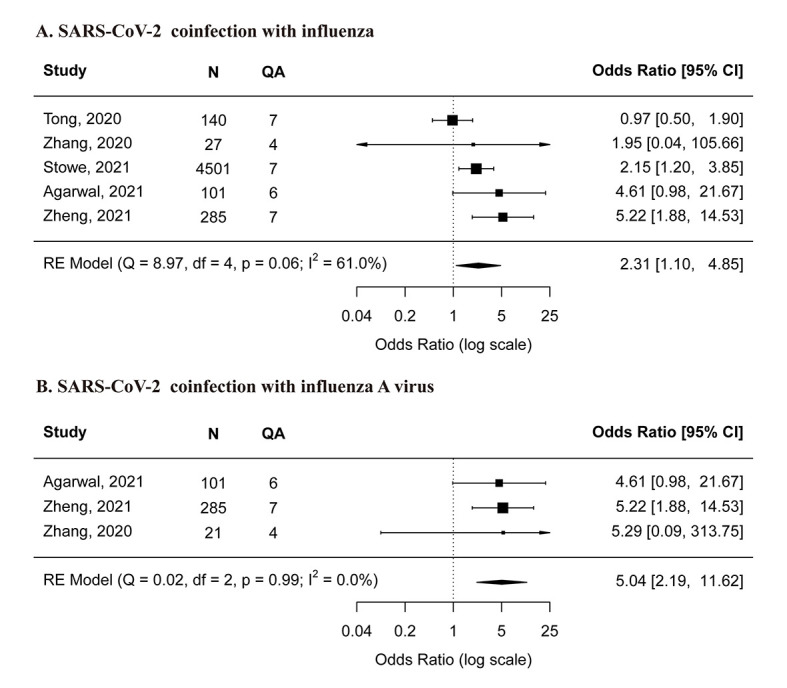
Comparison of risk for mechanical ventilation between SARS-CoV-2 mono-infection and A) SARS-CoV-2 co-infection with influenza, B) SARS-CoV-2 co-infection with influenza A virus. **Panel A** – SARS-CoV-2 co-infection with influenza. **Panel B** – SARS-CoV-2 co-infection with influenza A virus. N – the total number of SARS-CoV-2 coinfected and mono-infected patients, QA – the score of quality assessment.

Based on the meta-analysis results, SARS-CoV-2 co-infection with influenza was found to be associated with a higher risk of mechanical ventilation as compared to SARS-CoV-2 mono-infection (OR = 2.31, 95% CI = 1.10-4.85) (Figure 3, panel A). SARS-CoV-2 co-infection with influenza A virus was also associated with a higher risk mechanical ventilation (OR = 5.04, 95% CI = 2.19-11.62) (Figure 3, panel B). After excluding low-quality studies, a similar meta-estimate was observed (Figure S2, panel B in the **Online Supplementary Document**). Results from two high-quality studies [19,25] using multivariable models were consistent with our meta-estimates although another high-quality study [17] showed no significant difference in receiving mechanical ventilation between the two groups.

Regarding the SARS-CoV-2 co-infection with RSV, one moderate-quality study [27] indicated that the co-infection was not associated with increased risk of mechanical ventilation (OR = 5.00, 95% CI = 0.27-93.96) in children under two years old. The other low-quality [22] study reported no patients receiving mechanical ventilation in either group.

### Co-infection and risk of ICU

Six studies [18,19,22,24,25,27] compared the utilisation of intensive care between mono-infection and co-infection (all but one [27] on influenza and two [22,27] on RSV co-infection).

**Figure 4:**
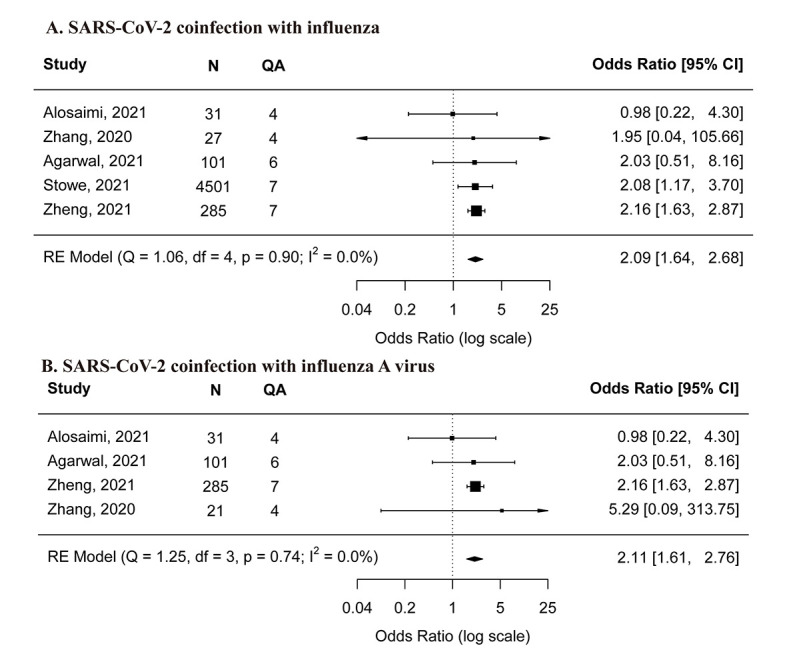
Comparison of risk for ICU admission between SARS-CoV-2 mono-infection and A) SARS-CoV-2 co-infection with influenza, B) SARS-CoV-2 co-infection with influenza A virus. **Panel A** – SARS-CoV-2 co-infection with influenza. **Panel B** – SARS-CoV-2 co-infection with influenza A virus. N – the total number of SARS-CoV-2 coinfected and mono-infected patients, QA – the score of quality assessment.

Based on the meta-analysis results, SARS-CoV-2 co-infection with influenza was associated with a higher risk of ICU admission compared with SARS-CoV-2 mono-infection (OR = 2.09, 95% CI = 1.64-2.68) (**Figure 4**, panel A). The sensitivity analysis revealed a similar meta-estimate when excluding low-quality studies (Figure S2, panel C in the **Online Supplementary Document**). SARS-CoV-2 co-infection with influenza A virus was also associated with a higher risk of ICU admission (OR = 2.11, 95% CI = 1.61-2.76) (**Figure 4**, panel B). Results from the two high-quality studies [19,25] were in accordance with the meta-analysis results.

Two studies [22,27] investigated the SARS-CoV-2 co-infection with RSV. One moderate-quality study [27] found no significant difference in ICU admission (OR = 2.40, 95% CI = 0.18-31.88) between mono- and co-infection groups. Another low-quality study [22] reported no patients being admitted to ICU.

### Co-infection and risk of death

All studies included in our review reported the proportion of deaths in mono- and co-infection groups (all but one [27] on influenza and three [22,23,27] on RSV co-infection).

**Figure 5:**
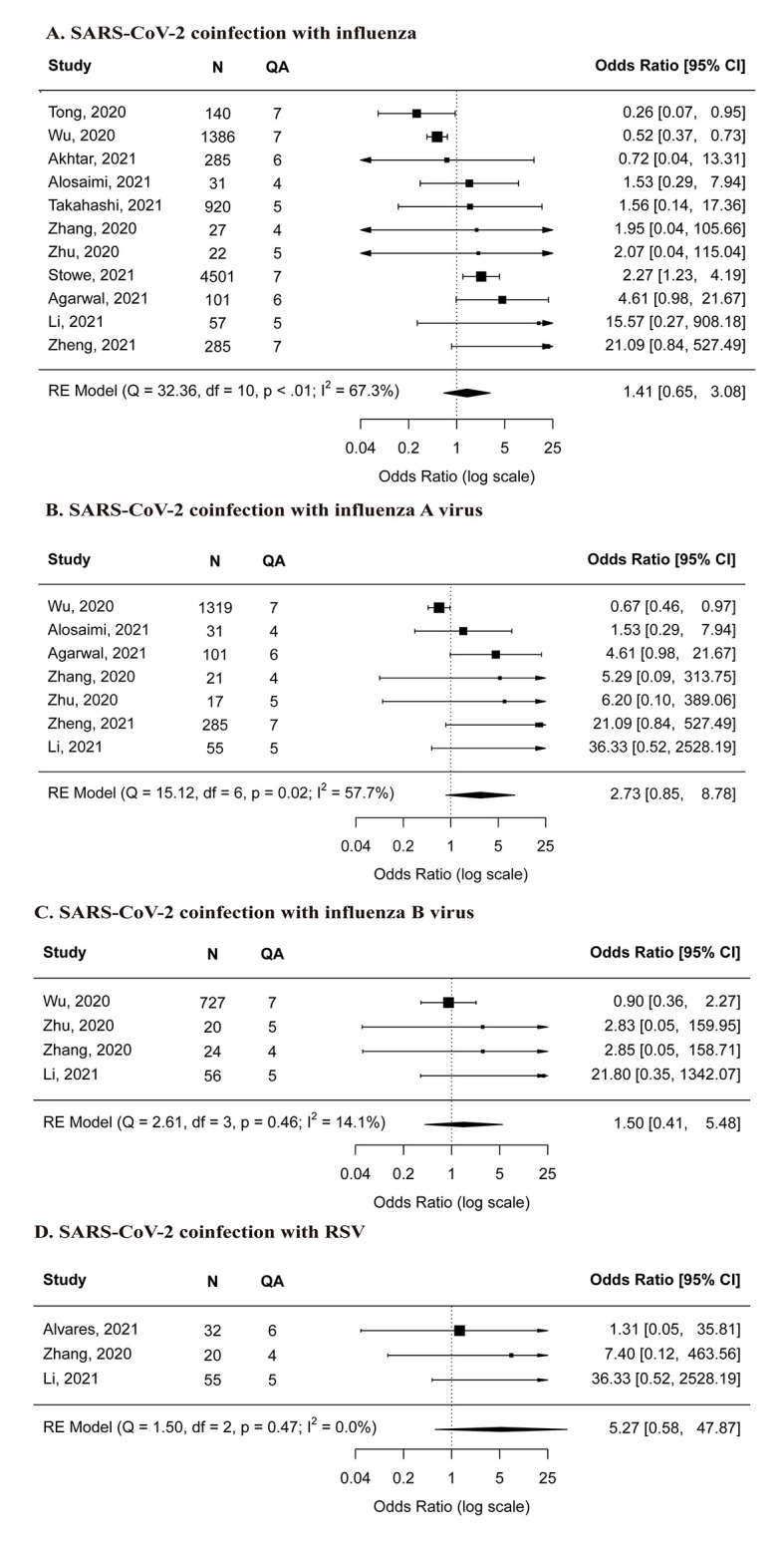
Comparison of risk for death between SARS-CoV-2 mono-infection and A) SARS-CoV-2 co-infection with influenza, B) SARS-CoV-2 co-infection with influenza A virus, C) SARS-CoV-2 co-infection with influenza B virus, D) SARS-CoV-2 co-infection with RSV. **Panel A** – SARS-CoV-2 co-infection with influenza. **Panel B** – SARS-CoV-2 co-infection with influenza A virus. **Panel C** – SARS-CoV-2 co-infection with influenza B virus. **Panel D** – SARS-CoV-2 co-infection with RSV. N – the total number of SARS-CoV-2 coinfected and mono-infected patients, QA – the score of quality assessment.

The meta-analysis showed that co-infection with SARS-CoV-2 and influenza was not associated with increased risk of death compared with SARS-CoV-2 mono-infection (OR = 1.41, 95% CI = 0.65-3.08) (**Figure 5**, panel A). Sensitivity analyses that excluded studies with small sample size and low-quality studies showed similar meta-estimates (Figure S1, panel B and Figure S2, panel D in the **Online Supplementary Document**). Similar results were also found in subgroup analysis by co-infection of influenza A and B virus (**Figure 5**, panel B and panel C). Findings from high-quality studies showed contrasting results: two studies [16,17] reported decreased risk of death in co-infection group (OR = 0.51, 95% CI = 0.36-0.73; OR = 0.26, 95% CI = 0.07-0.95, respectively) whereas another study [19] reported increased risk of death (OR = 2.27, 95% CI = 1.23-4.19); in addition, another two studies [24,25] (moderate-quality and high-quality, respectively) reported no differences in risk of death between the two groups (OR = 4.61, 95% CI = 0.98-21.67; OR = 21.09, 95% CI = 0.84-527.66, respectively).

With respect to SARS-CoV-2 co-infection with RSV, three lower-quality studies [22,23,27] reported very small number of co-infected patients (range = 1-6). Our meta-analysis results suggested that no significant association was found between the co-infection status and death (OR = 5.27, 95% CI = 0.58-47.87) (**Figure** 5, panel D).

## DISCUSSION

This systematic review and meta-analysis included 955 laboratory-confirmed COVID-19 patients co-infected with influenza or RSV and 6907 patients infected with SARS-CoV-2 alone from 12 retrospective observational studies. We found that co-infection with influenza was associated with a 2-fold increase in the risk for ICU admission and for mechanical ventilation among COVID-19 patients, while evidence was limited on the role of RSV co-infection. Co-infection with influenza did not seem to increase the risk of death in COVID-19 patients.

The 2-fold increase in the risk of ICU admission and receiving mechanical ventilation in COVID-19 patients coinfected with influenza could have important implications for the clinical management. COVID-19 patients who received influenza positive tests before or upon admission might benefit from early interventions that could prevent or slow disease deterioration. This also highlights the importance of influenza vaccination program that might have been suspended because of the COVID-19 pandemic [28].

Earlier systematic reviews that compared co-infections with any viruses (rather than co-infection with influenza as used in this study) with mono-infection among COVID-19 patients did not observe any statistical differences in ICU admission between the co-infection and mono-infection groups [29-31]. This suggests that the increased risk of ICU admission in the co-infection group observed in our study was likely to be influenza-specific. One of the possible explanations is that influenza co-infection could predispose patients to secondary bacterial infections, which could lead to more severe clinical outcomes [32,33]. This explanation is supported by a recently published study in Israel that observed substantial reductions in pneumococcal diseases in young children during the COVID-19 pandemic; the authors showed that the reductions in pneumococcal and pneumococcus-associated diseases were not predominantly related to reduced pneumococcal carriage and density but were strongly associated with the disappearance of specific respiratory viruses such as influenza [34]. The role of influenza co-infection in the increased severity of COVID-19 patients was also supported by influenza probe studies. In a large Brazilian cohort study of over 53 000 COVID-19 patients, those who received a recent influenza vaccine had 7% lower risks of requiring intensive care treatment (95% CI = 2%-13%) and 17% lower risks of requiring invasive respiratory support (95% CI = 12%-23%) although this finding should be interpreted in the context of not controlling for residual confounding [35]. Moreover, a recently published animal model study found that simultaneous or sequential co-infection by SARS-CoV-2 and the influenza A(H1N1)pdm09 strain caused more severe disease than mono-infection by either virus in hamsters [36].

However, a systematic review and meta-analysis by Guan et al. reported that influenza co-infection lowered the risk for critical outcomes (composite outcomes including any of shock, being admitted to ICU, and requiring ventilatory support), with a pooled OR of 0.64 (95% CI = 0.43-0.97) based on five studies [37]. As this was contrary to our findings, we closely compared the methodology and the extractions between our study and that by Guan et al.; besides the use of composite outcomes as mentioned above (which differed from our study), the meta-estimate by Guan et al. was largely driven by a preprint that only had univariate ORs available for extraction. Moreover, there seemed to be a discrepancy in the ORs extracted by Guan et al. – the included study by Stowe et al. [19] reported (through multivariate analysis) that COVID-19 patients with influenza co-infection were around twice as likely to be ventilated (OR = 2.15, 95% CI = 1.20-3.84) or to be admitted to ICU (OR = 2.08, 95% CI = 1.17-3.70), while Guan et al. extracted an OR of 0.91 (95% CI = 0.41-2.02). After removing the preprint and correcting the extraction from the included studies by Guan et al., their updated OR increased to 1.76 (95% CI = 1.06-2.92), which re-confirmed the robustness of the meta-estimates in our study (details are provided in Figure S4 in the **Online Supplementary Document**).

Our meta-analysis revealed that co-infection with SARS-CoV-2 and influenza had no observable effects on the overall mortality in both the main and the two sensitivity analyses (ie, excluding studies with small sample size and excluding low-quality studies). Nonetheless, these findings did not indicate that co-infection of influenza did not increase the risks of mortality. We could not rule out type II error (ie, false negative) due to the limited statistical power; most studies had less than 50 COVID-19 patients with co-infections and the median number of deaths was 1 (IQR = 0-4). Moreover, the point estimates from the main and sensitivity analyses were consistently above 1, favouring the association between influenza co-infection and mortality.

Similar to influenza co-infection, no effects on the overall mortality were observed for RSV co-infection, but this was only based on three small studies. Our review highlighted the gaps in the knowledge on the role of RSV co-infection in COVID-19 disease severity. A retrospective study among six children’s hospitals in the United States revealed that one in six COVID-19 inpatients under 18 years old had viral co-infections, with two thirds of viral co-infections being RSV co-infections; the proportion of viral co-infections was even higher in infants under one year – one in three infants hospitalized for COVID-19 had viral co-infections, with almost three quarters of them having RSV co-infections [38]. The substantial proportion of RSV co-infection among paediatric COVID-19 patients calls for further research on the possible synergistic effects of the RSV and SARS-CoV-2 in this vulnerable age group.

At the time of writing, a large-scale study from the UK (including about 7000 COVID-19 patients) was published, which reported that that influenza co-infection was associated with increased odds of receiving invasive mechanical ventilation among COVID-19 patients (OR = 4.1, 95% CI = 2.0-8.5) in a multivariable regression analysis, further confirming the findings of our study [39]. The authors of that study did not find any significant associations for co-infection with other viruses including adenovirus and RSV, confirming our speculation that the association on viral co-infection and COVID-19 clinical severity is specific to influenza. Ad hoc inclusion of the estimates from that study did not substantially change our meta-estimates (Table S2 in the **Online Supplementary Document**).

We acknowledge some study limitations. First, several factors could contribute to heterogeneity in our findings, such as study population (including age and sex), case definition, specimen sources, sample size, laboratory method, and statistical method; we were unable to explicitly account for these variations. We were also unable to account for bacterial co-infection in the analysis due to the absence of data that had reliable laboratory confirmation on viral and bacterial infections. Additionally, whether the subjects of primary studies were vaccinated against influenza prior to recruitment was almost unknown, which may affect the severity of the viral infection. To explore heterogeneity, sensitivity analyses excluding studies with small sample sizes and excluding those low-quality studies were performed. However, we could not carry out subgroups analyses by aforementioned factors due to the limited data. In this review, there were only four studies applying multivariate analyses to adjust for common confounders between co-infections and severity outcomes. Although restricting to these high-quality studies did not substantially change any of our findings, this highlighted the lack of high-quality studies on the role of co-infections and COVID-19 disease severity. Second, compared with other outcomes, mortality as the proxy for disease severity was more likely to be affected by variations in clinical treatment, which could confound the estimates if treatment differed by status of co-infection. Therefore, there is a need for a larger well-designed study further investigating the impact of influenza co-infection on overall mortality. Third, all included studies were conducted in 2020, when the activity of influenza and RSV was gradually decreasing; it remains unknown whether the resurgence of influenza and RSV could modify the association reported in this review. In summary, our results should still be interpreted with caution and considered as preliminary findings due to significant heterogeneity among studies and limited sample size.

## CONCLUSIONS

Despite its limitations, this systematic review and meta-analysis comprehensively summarized an extensive literature search and critically appraised and synthesised existing evidence on the role of influenza and RSV co-infection in the clinical severity of COVID-19 patients. While our study highlights the gaps in research on this topic, particularly for RSV, existing evidence suggests that influenza co-infection could increase the disease severity of COVID-19 patients, which could have important implications for clinical management, as well as influenza vaccination campaigns in the context of resurgence of influenza among other respiratory viruses in the post-COVID-19 era.

## Additional material


Online Supplementary Document
