# Supplementary material for: The role of respiratory co-infection with influenza or respiratory syncytial virus in the clinical severity of COVID-19 patients: A systematic review and meta-analysis
Source: J Glob Health. 2022 Sep 17;12:05040. doi: 10.7189/jogh.12.05040 (PMC9480863; doi:10.7189/jogh.12.05040)
Supplement: Online Supplementary Document [file jogh-12-05040-s001.pdf]

ONLINE SUPPLEMENTARY DOCUMENT

Title: The role of respiratory co-infection with influenza or respiratory syncytial virus in the clinical severity of COVID-19 patients: a systematic review and meta-analysis

Authors: Bingbing Cong[1]; Shuyu Deng[1]; Xin Wang[2]; You Li[1]*

Affiliations:

[1] Department of Epidemiology, School of Public Health, Nanjing Medical University, Nanjing, 211166, China

[2] Department of Biostatistics, School of Public Health, Nanjing Medical University, Nanjing, 211166, China

* Correspondence to Prof. You Li (you.li@njmu.edu.cn), Department of Epidemiology, School of Public Health, Nanjing Medical University, Nanjing, 211166, China

Contents

Text S1. PRISMA 2020 Checklist 4

Text S2. Synthesis without meta-analysis in systematic reviews (SWiM) reporting guideline .. 7

Text S3. Search strategy 9

Medline (Ovid) 9

Embase (Ovid) 10

Web of science 11

WHO COVID-19 database 12

CNKI (search terms are in Chinese) 12

Sinomond (search terms are in Chinese) 12

CqVip (search terms are in Chinese) 13

WanFang (search terms are in Chinese) 13

Table S1. Quality assessment of included studies 14

Table S2. Comparison of meta-analysis results before and after including a large-scale 16

multicenter study* that was published at the time of manuscript writing 16

Figure S1. Forest plots for meta-analysis of outcomes excluding studies of the number of subjects of co-infected group or mono-infected group ≤ 5 (only available for SARS-CoV-2 . 17

coinfection with influenza vs SARS-CoV-2 mono-infection) 17

Figure S2. Forest plots for meta-analysis of outcomes excluding low-quality studies (only available for SARS-CoV-2 coinfection with influenza vs SARS-CoV-2 mono-infection) 18

Figure S3. Funnel plots of death outcome (SARS-CoV-2 coinfection with influenza virus vs 20

SARS-CoV-2 mono-infection) 20

Figure S4. Forest plots for meta-analysis of critical outcomes and SARS-CoV-2 coinfection 22

with influenza in different scenarios (based on the review by Guan et al.) 22

Text S1. PRISMA 2020 Checklist

Section and Topic	Item #	Checklist item	Location where item is reported
TITLE			
Title	1	Identify the report as a systematic review.	1
ABSTRACT			
Abstract	2	See the PRISMA 2020 for Abstracts checklist.	2-3
INTRODUCTION			
Rationale	3	Describe the rationale for the review in the context of existing knowledge.	3
Objectives	4	Provide an explicit statement of the objective(s) or question(s) the review addresses.	3-4
METHODS			
Eligibility criteria	5	Specify the inclusion and exclusion criteria for the review and how studies were grouped for the syntheses.	4-5
Information sources	6	Specify all databases, registers, websites, organisations, reference lists and other sources searched or consulted to identify studies. Specify the date when each source was last searched or consulted.	4-5
Search strategy	7	present the full search strategies for all databases, registers and websites, including any filters and limits used.	Appendix pp 7-10
Selection process	8	Specify the methods used to decide whether a study met the inclusion criteria of the review, including how many reviewers screened each record and each report retrieved, whether they worked independently, and if applicable, details of automation tools used in the process.	4-5, 20
Data collection process	9	Specify the methods used to collect data from reports, including how many reviewers collected data from each report, whether they worked independently, any processes for obtaining or confirming data from study investigators, and if applicable, details of automation tools used in the process.	4-5

Data items	10a	List and define all outcomes for which data were sought. Specify whether all results that were compatible with each outcome domain in each study were sought (e.g. for all measures, time points, analyses), and if not, the methods used to decide which results to collect.	4-5
	10b	List and define all other variables for which data were sought (e.g. participant and intervention characteristics, funding sources). Describe any assumptions made about any missing or unclear information.	4-5
Study risk of bias assessment	11	Specify the methods used to assess risk of bias in the included studies, including details of the tool(s) used, how many reviewers assessed each study and whether they worked independently, and if applicable, details of automation tools used	5-6

Section and Topic	Item #	Checklist item	Location where item is reported
		in the process.	
Effect measures	12	Specify for each outcome the effect measure(s) (e.g. risk ratio, mean difference) used in the synthesis or presentation of results.	6-7
Synthesis methods	13a	Describe the processes used to decide which studies were eligible for each synthesis (e.g. tabulating the study intervention characteristics and comparing against the planned groups for each synthesis (item #5)).	6-7
	13b	Describe any methods required to prepare the data for presentation or synthesis, such as handling of missing summary statistics, or data conversions.	6-7
	13c	Describe any methods used to tabulate or visually display results of individual studies and syntheses.	6-7
	13d	Describe any methods used to synthesize results and provide a rationale for the choice(s). If meta-analysis was performed, describe the model(s), method(s) to identify the presence and extent of statistical heterogeneity, and software package(s) used.	6-7
	13e	Describe any methods used to explore possible causes of heterogeneity among study results (e.g. subgroup analysis, metaregression).	6-7
	13f	Describe any sensitivity analyses conducted to assess robustness of the synthesized results.	6-7
Reporting bias assessment	14	Describe any methods used to assess risk of bias due to missing results in a synthesis (arising from reporting biases).	6-7
Certainty assessment	15	Describe any methods used to assess certainty (or confidence) in the body of evidence for an outcome.	6-7

RESULTS			
Study selection	16a	Describe the results of the search and selection process, from the number of records identified in the search to the number of studies included in the review, ideally using a flow diagram.	7
	16b	Cite studies that might appear to meet the inclusion criteria, but which were excluded, and explain why they were excluded.	7
Study characteristics	17	Cite each included study and present its characteristics.	7-8, 21
Risk of bias in studies	18	Present assessments of risk of bias for each included study.	8, appendix pp 11
Results of individual studies	19	For all outcomes, present, for each study: (a) summary statistics for each group (where appropriate) and (b) an effect estimate and its precision (e.g. confidence/credible interval), ideally using structured tables or plots.	7-8, 21
Results of syntheses	20a	For each synthesis, briefly summarise the characteristics and risk of bias among contributing studies.	Appendix pp 11

Section and Topic	Item #	Checklist item	Location where item is reported
	20b	Present results of all statistical syntheses conducted. If meta-analysis was done, present for each the summary estimate and its precision (e.g. confidence/credible interval) and measures of statistical heterogeneity. If comparing groups, describe the direction of the effect.	8-11, 23-28
	20c	Present results of all investigations of possible causes of heterogeneity among study results.	7-8, 15
	20d	Present results of all sensitivity analyses conducted to assess the robustness of the synthesized results.	appendix pp 12-14
Reporting biases	21	Present assessments of risk of bias due to missing results (arising from reporting biases) for each synthesis assessed.	Appendix pp 15
Certainty of evidence	22	Present assessments of certainty (or confidence) in the body of evidence for each outcome assessed.	Appendix pp 11
DISCUSSION			
Discussion	23a	Provide a general interpretation of the results in the context of other evidence.	11-14
	23b	Discuss any limitations of the evidence included in the review.	12-14
	23c	Discuss any limitations of the review processes used.	15

	23d	Discuss implications of the results for practice, policy, and future research.	12, 15
OTHER INFORMATION			
Registration and protocol	24a	Provide registration information for the review, including register name and registration number, or state that the review was not registered.	4
	24b	Indicate where the review protocol can be accessed, or state that a protocol was not prepared.	4
	24c	Describe and explain any amendments to information provided at registration or in the protocol.	4
Support	25	Describe sources of financial or non-financial support for the review, and the role of the funders or sponsors in the review.	16
Competing interests	26	Declare any competing interests of review authors.	16
Availability of data, code and other materials	27	Report which of the following are publicly available and where they can be found: template data collection forms; data extracted from included studies; data used for all analyses; analytic code; any other materials used in the review.	16

Text S2. Synthesis without meta-analysis in systematic reviews (SWiM) reporting guideline

Synthesis Without Meta-analysis (SWiM) items: SWiM is intended to complement and be used as an extension to PRISMA

SWiM reporting item	Item description	Page in manuscript where item is reported	Other*
**Methods**			
1 Grouping studies for synthesis	1a) Provide a description of, and rationale for, the groups used in the synthesis (eg, groupings of populations, interventions, outcomes, study design)<br>1b) Detail and provide rationale for any changes made subsequent to the protocol in the groups used in the synthesis	4-5	
2 Describe the standardised metric and transformation methods used	Describe the standardised metric for each outcome. Explain why the metric(s) was chosen and describe any methods used to transform the intervention effects, as reported in the study, to the standardised metric, citing any methodological guidance consulted	4-5	
3 Describe the synthesis methods	Describe and justify the methods used to synthesise the effects for each outcome when it was not possible to undertake a meta-analysis of effect estimates	6-7	
4 Criteria used to prioritise results for summary and synthesis	Where applicable, provide the criteria used, with supporting justification, to select the particular studies, or a particular study, for the main synthesis or to draw conclusions from the synthesis (eg, based on study design, risk of bias assessments, directness in relation to the review question)	6-7	

5 Investigation of heterogeneity in reported	State the method(s) used to examine heterogeneity in reported effects when it was not possible to undertake a meta-analysis of effect estimates and its extensions to investigate heterogeneity effects	6-7
6 Certainty of evidence	Describe the methods used to assess the certainty of the synthesis findings	6
7 Data presentation	Describe the graphical and tabular methods used to present the effects (eg, tables, forest plots, harvest plots) Specify key study characteristics (eg, study design, risk of bias) used to order the studies, in the text and any tables or graphs, clearly referencing the studies included	6-7 methods
**Results**		
8 Reporting results	For each comparison and outcome, provide a description of the synthesised findings and the certainty of the findings. Describe the result in language that is consistent with the question the synthesis addresses, and indicate which studies contribute to the synthesis	7-11, 20-25
**Discussion**		
9 Limitations of the synthesis	Report the limitations of the synthesis methods used and/or the groupings used in the synthesis and how these affect the conclusions that can be drawn in relation to the original review question	11-15

PRISMA=Preferred Reporting Items for Systematic Reviews and Meta-Analyses.
*If the information is not provided in the systematic review, give details of where this information is available (eg, protocol, other published papers (provide citation details), or website (provide the URL)).

Text S3. Search strategy

Medline (Ovid)

1. exp Respiratory Syncytial Virus Infections/ or exp Respiratory Syncytial Viruses/ or Respiratory syncytial virus*.mp. or exp Respiratory Syncytial Virus, Human/
2. (RSV or respiratory syncytial).mp.
3. exp Bronchiolitis/ or bronchiolitis.mp. or exp Bronchiolitis,viral/
4. exp Pneumonia/ or pneumonia.mp. or exp Pneumonia,viral/
5. (lower respiratory tract infection* or lower respiratory infection* or respiratory infection*).mp. or exp respiratory tract infections/

6.exp Influenza, Human/ or influenza.mp. or flu.mp.

7. 2019-nCoV.mp. or *SARS-CoV-2/
8. (Severe Acute Respiratory Syndrome Coronavirus 2 or COVID-19 Virus* or 2019 Novel Coronavirus* or Coronavirus Disease 2019 Virus* or Novel Coronavirus*, or 2019 SARS Coronavirus 2).mp.
9. exp Coinfection/ or coinfection*.mp.
10. exp Superinfection/ or superinfection*.mp.
11. (mixed infection* or multiple infection* or codetection* or secondary infection* or polymicrobial infection*).mp.
12. exp Oxygen Inhalation Therapy/ or oxygen therapy.mp. or exp Respiratory Insufficiency/
13. exp Hypoxia/ or supplemental oxygen.mp. or oxygen requirement.mp.
14. mechanical ventilation.mp. or exp Respiration, Artificial/
15. exp mortality/ or exp death/ or death.mp. or mortality.mp.
16. exp Intensive Care Units/ or exp Critical Care/ or intensive care unit*.mp.

17. (intensive care or ICU).mp.

18. 1 or 2 or 3 or 4 or 5 or 6

19. 7 or 8

20. 9 or 10 or 11

21. 12 or 13 or 14 or 15 or 16 or 17

22. 18 and 19 and 20 and 21

23. limit 22 to yr="2020 -2021"

Embase (Ovid)

1. exp Respiratory Syncytial Virus Infection/ or exp Respiratory syncytial pneumovirus/ or

Respiratory syncytial virus*.mp. or exp Human Respiratory Syncytial Virus/

2. (RSV or respiratory syncytial).mp.

3. exp Bronchiolitis/ or bronchiolitis.mp. or exp viral Bronchiolitis/

4. exp Pneumonia/ or exp Community acquired pneumonia/ or exp Virus Pneumonia/ or exp viral Pneumonia/ or pneumonia.mp.

5. (lower respiratory tract infection* or lower respiratory infection* or respiratory infection*).mp. or exp respiratory tract infections/ or exp lower respiratory tract infection/

6. exp influenza/ or influenza.mp. or flu.mp.

7. 2019-nCoV.mp. or exp Severe Acute Respiratory Syndrome Coronavirus 2/ or exp

Coronavirus Disease 2019/

8. (Severe Acute Respiratory Syndrome Coronavirus 2 or COVID-19 Virus* or 2019 Novel

Coronavirus* or Coronavirus Disease 2019 Virus* or Novel Coronavirus*, or 2019 SARS

Coronavirus 2).mp.

9. exp Coinfection/ or coinfection*.mp.

10. exp Superinfection/ or superinfection*.mp.

11. (mixed infection* or multiple infection* or codetection* or secondary infection* or polymicrobial infection*).mp.

12. exp Oxygen Therapy/ or exp oxygen supply/ or oxygen therapy.mp. or exp Respiratory Failure/

13. exp Hypoxia/ or supplemental oxygen.mp. or oxygen requirement.mp.

14. mechanical ventilation.mp. or exp Artificial ventilation/

15. exp mortality/ or exp death/ or (mortality or death).mp.

16. exp Intensive Care Unit/ or exp Critical Care/ or intensive care unit*.mp.

17. (intensive care or ICU).mp.

18. 1 or 2 or 3 or 4 or 5 or 6

19. 7 or 8

20. 9 or 10 or 11

21. 12 or 13 or 14 or 15 or 16 or 17

22. 18 and 19 and 20 and 21

23. limit 22 to yr="2020 -2021"

Web of science

1.TS= (Respiratory Syncytial Virus Infection* OR Respiratory Syncytial Virus* OR Respiratory Syncytial Virus, Human OR RSV OR Bronchiolitis,viral OR Pneumonia,viral OR lower respiratory tract infection* OR Respiratory Tract Infection* OR Influenza, Human OR Influenza OR flu)

2. TS= (2019-nCoV OR SARS-CoV-2 OR COVID-19 OR Severe Acute Respiratory Syndrome Coronavirus 2 or COVID-19 Virus* OR 2019 Novel Coronavirus*)

3.TS= (Coinfection* OR Superinfection* OR mixed infection* OR multiple infection* OR codetection* OR dual infection*)

4.TS= (Oxygen Inhalation Therapy OR oxygen therapy OR Respiratory Insufficiency OR Respiratory Therapy OR Hypoxia OR mechanical ventilation OR Respiration,Artificial OR mortality OR death OR Intensive Care Unit* OR Critical Care OR intensive care OR ICU)

(((#1) AND #2) AND #3) AND #4

2020-01-01 to 2021-12-31

WHO COVID-19 database

((tw:(influenza)) OR (tw:(respiratory syncytial virus))) AND (tw:(coinfection*))

CNKI (search terms are in Chinese)

(篇关摘%呼吸道合胞病毒 + 合胞病毒 + RSV + 流行性感冒病毒 + 流感病毒)

AND (篇关摘%新冠病毒 + 新型冠状病毒 + 新冠肺炎)

Sinomond (search terms are in Chinese)

("呼吸道合胞病毒"[常用字段:智能] OR " 合胞病毒"[常用字段:智能] OR "RSV"[常用字段:智能] OR "流行性感冒病毒"[常用字段:智能] OR "流感病毒"[常用字段:智能]) AND(" 新冠病毒"[常用字段:智能] OR "新型冠状病毒"[常用字段:智能] OR "新冠肺炎"[常用字段:智能]) AND("共同感染"[常用字段:智能] OR "混合感染"[常用字段:智能] OR "同时感染"[常用字段:智能]) AND 2020-2021[日期]

CqVip (search terms are in Chinese)

(任意字段=流行性感冒病毒 + 流感病毒 +呼吸道合胞病毒 + 合胞病毒 + RSV

AND 任意字段=新冠病毒 + 新冠肺炎 +新型冠状病毒) AND 年份：2020-2021

WanFang (search terms are in Chinese)

(主题:(呼吸道合胞病毒 OR 合胞病毒 OR RSV OR 流行性感冒病毒 OR 流感病毒) and

主题:(新冠病毒 OR 新型冠状病毒 OR 新冠肺炎) and 主题:(共同感染 OR 混合

感染 OR 同时感染)) and Date:2020-2021

Table S1. Quality assessment of included studies

Study	Did the study address a clearly focused issue?	Were the subjects recruited in an acceptable way?	Was the exposure accurately measured to minimal bias?	Was the outcome accurately measured to minimal bias?	Have the authors used multivariable analysis model in the analysis?	Can the results be applied to the local population?	Do the results of this study fit with other available evidence?
Wu, 2020	yes	yes	yes	yes	yes	yes	yes
Alvares, 2021	yes	yes	yes	yes	no	yes	yes
Tong, 2020	yes	yes	yes	yes	yes	yes	yes
Alosaimi,2021	yes	no	yes	yes	no	no	yes
Stowe, 2021	yes	yes	yes	yes	yes	yes	yes
Takahashi,2021	no	yes	yes	yes	no	yes	yes
Zhu, 2020	no	yes	yes	yes	no	yes	yes
Zhang, 2020	no	yes	yes	no	no	yes	yes
Li, 2021	no	yes	yes	yes	no	yes	yes
Agarwal,2021	yes	yes	yes	yes	no	yes	yes
Zheng, 2021	yes	yes	yes	yes	yes	yes	yes
Akhtar, 2021	yes	yes	yes	yes	no	yes	yes

Table S2. Comparison of meta-analysis results before and after including a large-scale multicenter study* that was published at the time of manuscript writing

Co-infection type	Mechanical ventilation		Deaths	
	Before inclusion	After inclusion	Before inclusion	After inclusion
RSV co-infection	—	—	OR: 5.27 (95% CI: 0.58-47.87)	OR: 1.72 (95% CI: 0.29-10.18)
Influenza co-infection	OR: 2.31 (95% CI: 1.10-4.85)	OR: 2.61 (95% CI: 1.39-4.90)	OR: 1.41 (95% CI: 0.65-3.08)	OR: 1.50 (95% CI: 0.75-2.98)

*Swets MC et al. Lancet 2022.

Figure S1. Forest plots for meta-analysis of outcomes excluding studies of the number of subjects of co-infected group or mono-infected group ≤ 5 (only available for SARS-CoV-2 coinfection with influenza vs SARS-CoV-2 mono-infection)

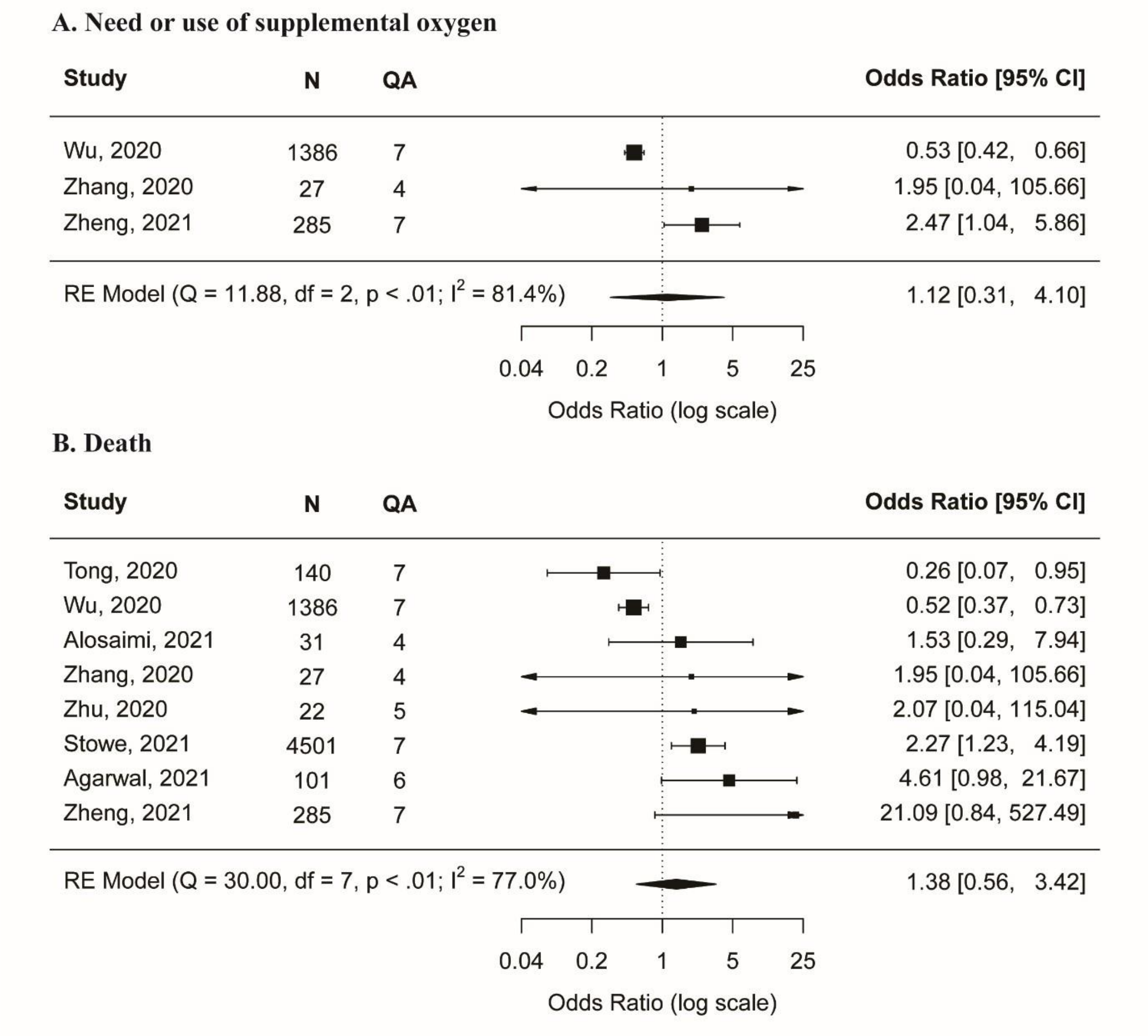

Note: N-the total number of SARS-CoV-2 coinfected and mono-infected patients; QA-the score of quality assessment.

Figure S2. Forest plots for meta-analysis of outcomes excluding low-quality studies (only available for SARS-CoV-2 coinfection with influenza vs SARS-CoV-2 monoinfection)

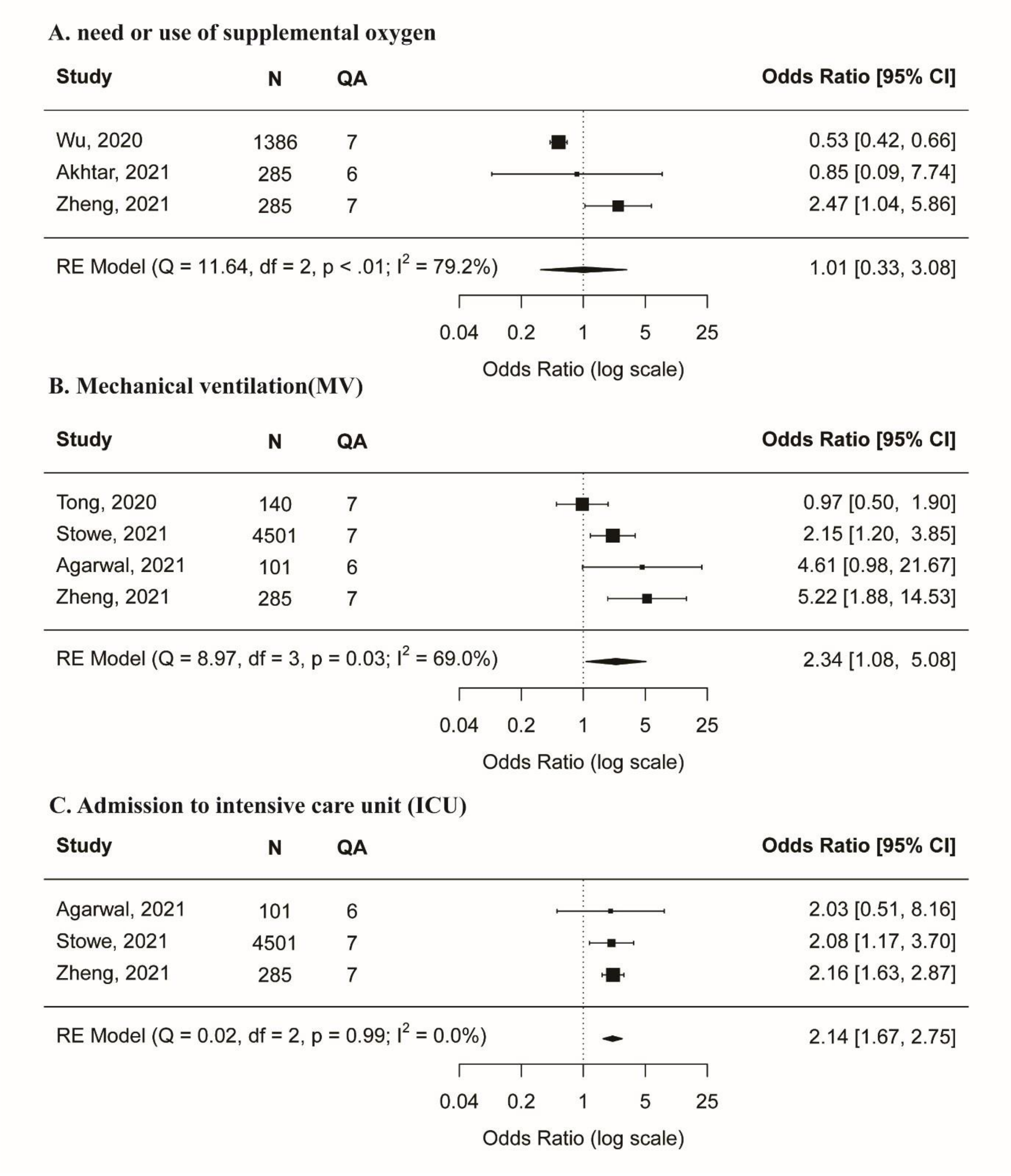

Figure continues on next page

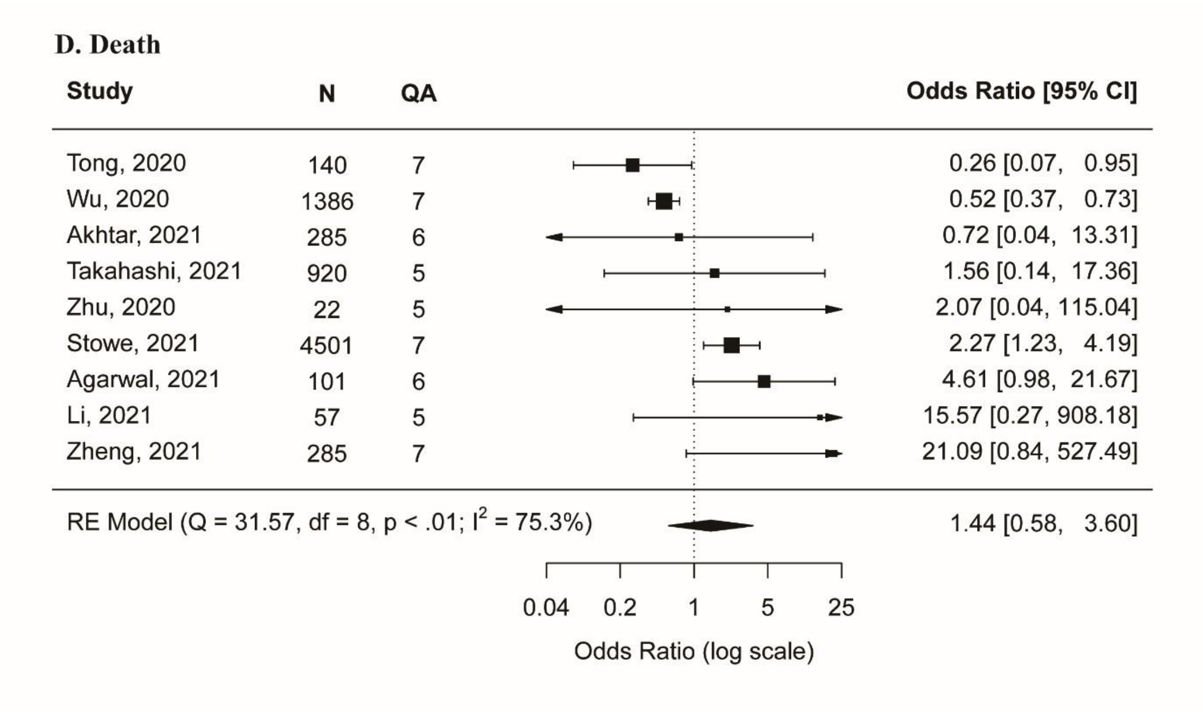

Note: N-the total number of SARS-CoV-2 coinfected and mono-infected patients; QA-the score of quality assessment.

Figure S3. Funnel plots of death outcome (SARS-CoV-2 coinfection with influenza virus vs SARS-CoV-2 mono-infection)

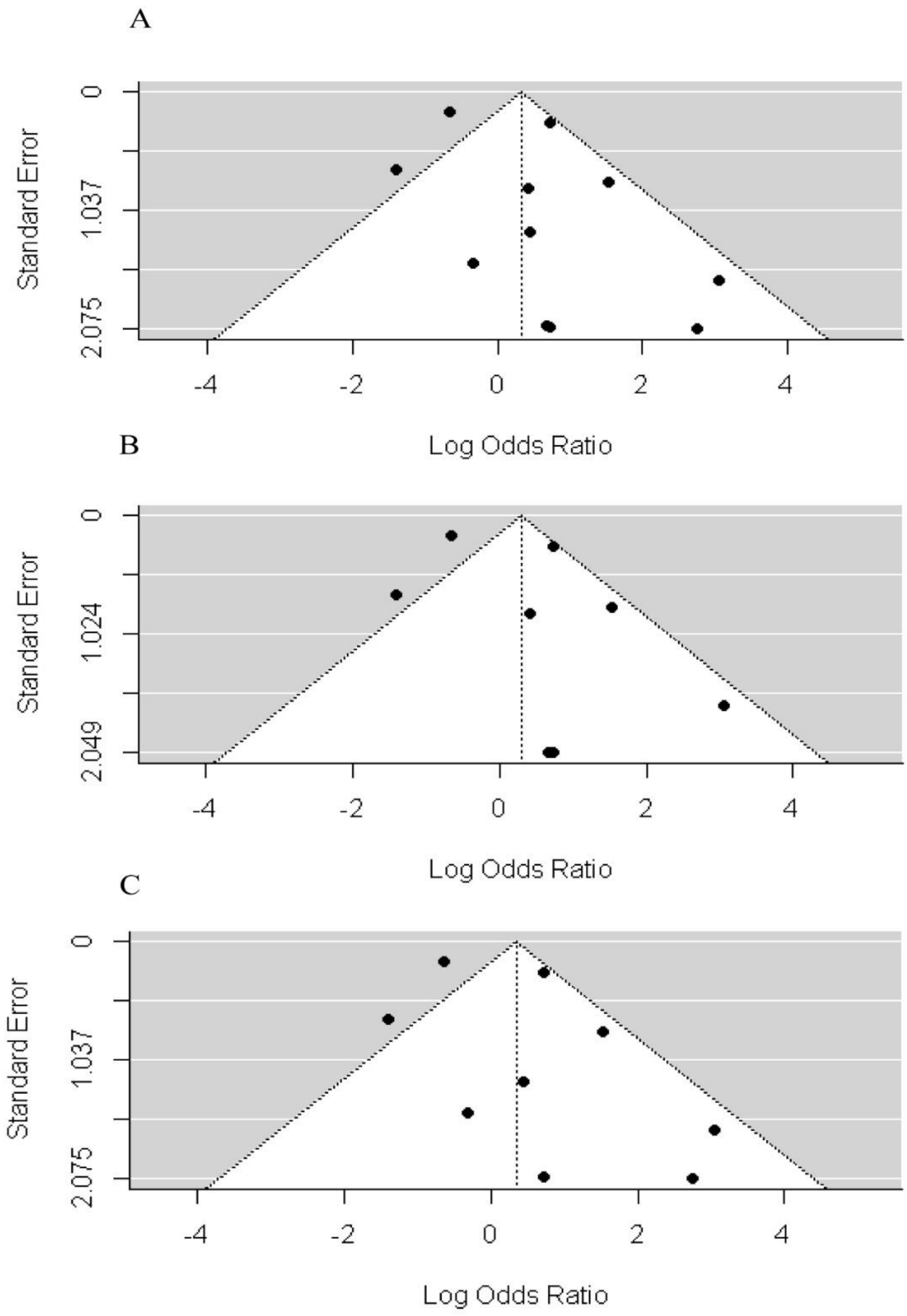

Note that figure (A), (B) and (C) indicates main analysis, sensitivity analysis when excluding studies with small sample size and sensitivity analysis when excluding low-quality studies, respectively.

Figure S4. Forest plots for meta-analysis of critical outcomes and SARS-CoV-2 coinfection with influenza in different scenarios (based on the review by Guan et al.)

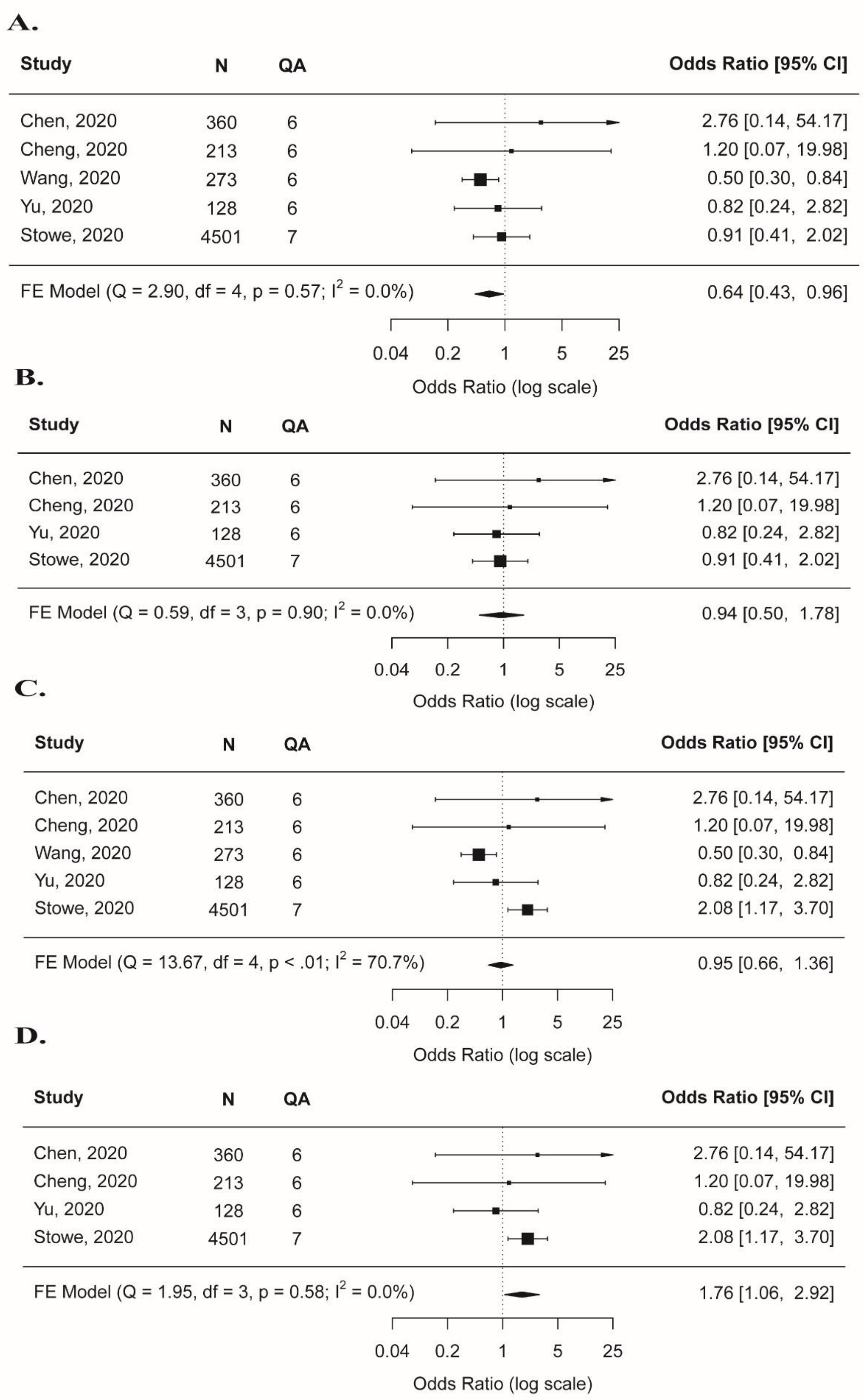

A.
Study
N
QA
Odds Ratio [95% CI]
Chen, 2020 360 6 2.76 [0.14, 54.17]
Cheng, 2020 213 6 1.20 [0.07, 19.98]
Wang, 2020 273 6 0.50 [0.30, 0.84]
Yu, 2020 128 6 0.82 [0.24, 2.82]
Stowe, 2020 4501 7 0.91 [0.41, 2.02]
FE Model (Q = 2.90, df = 4, p = 0.57; I^2 = 0.0%) 0.64 [0.43, 0.96]
0.04 0.2 1 5 25
Odds Ratio (log scale)
B.
Study
N
QA
Odds Ratio [95% CI]
Chen, 2020 360 6 2.76 [0.14, 54.17]
Cheng, 2020 213 6 1.20 [0.07, 19.98]
Yu, 2020 128 6 0.82 [0.24, 2.82]
Stowe, 2020 4501 7 0.91 [0.41, 2.02]
FE Model (Q = 0.59, df = 3, p = 0.90; I^2 = 0.0%) 0.94 [0.50, 1.78]
0.04 0.2 1 5 25
Odds Ratio (log scale)
C.
Study
N
QA
Odds Ratio [95% CI]
Chen, 2020 360 6 2.76 [0.14, 54.17]
Cheng, 2020 213 6 1.20 [0.07, 19.98]
Wang, 2020 273 6 0.50 [0.30, 0.84]
Yu, 2020 128 6 0.82 [0.24, 2.82]
Stowe, 2020 4501 7 2.08 [1.17, 3.70]
FE Model (Q = 13.67, df = 4, p < .01; I^2 = 70.7%) 0.95 [0.66, 1.36]
0.04 0.2 1 5 25
Odds Ratio (log scale)
D.
Study
N
QA
Odds Ratio [95% CI]
Chen, 2020 360 6 2.76 [0.14, 54.17]
Cheng, 2020 213 6 1.20 [0.07, 19.98]
Yu, 2020 128 6 0.82 [0.24, 2.82]
Stowe, 2020 4501 7 2.08 [1.17, 3.70]
FE Model (Q = 1.95, df = 3, p = 0.58; I^2 = 0.0%) 1.76 [1.06, 2.92]
0.04 0.2 1 5 25
Odds Ratio (log scale)

Note: A). results replicated from Guan and colleagues; B). results when removing the preprint only extracting univariate OR for analysis; C). results when extracting multivariate OR (i.e., correcting the extraction) for analysis; D). results when removing the preprint and correcting the extraction simultaneously. N-the total number of SARS-CoV-2 coinfected and monoinfected patients; QA-the score of quality assessment. The quality assessment was conducted using the criteria of the present review.
